# Activity-Based Probes to Utilize the Proteolytic Activity of Cathepsin G in Biological Samples

**DOI:** 10.3389/fchem.2021.628295

**Published:** 2021-02-25

**Authors:** Timo Burster, Fabian Gärtner, Uwe Knippschild, Anuar Zhanapiya

**Affiliations:** ^1^ Department of Biology, School of Sciences and Humanities, Nazarbayev University, Nur-Sultan, Kazakhstan; ^2^ Department of General and Visceral Surgery, Surgery Center, Ulm University Hospital, Ulm, Germany

**Keywords:** activity-based probes, cathepsin G, flow cytometry, CyTOF/mass cytometry, immune cells, serine proteases

## Abstract

Neutrophils, migrating to the site of infection, are able to release serine proteases after being activated. These serine proteases comprise cathepsin G (CatG), neutrophil elastase protease 3 (PR3), and neutrophil serine protease 4 (NSP4). A disadvantage of the uncontrolled proteolytic activity of proteases is the outcome of various human diseases, including cardiovascular diseases, thrombosis, and autoimmune diseases. Activity-based probes (ABPs) are used to determine the proteolytic activity of proteases, containing a set of three essential elements: Warhead, recognition sequence, and the reporter tag for detection of the covalent enzyme activity–based probe complex. Here, we summarize the latest findings of ABP-mediated detection of proteases in both locations intracellularly and on the cell surface of cells, thereby focusing on CatG. Particularly, application of ABPs in regular flow cytometry, imaging flow cytometry, and mass cytometry by time-of-flight (CyTOF) approaches is advantageous when distinguishing between immune cell subsets. ABPs can be included in a vast panel of markers to detect proteolytic activity and determine whether proteases are properly regulated during medication. The use of ABPs as a detection tool opens the possibility to interfere with uncontrolled proteolytic activity of proteases by employing protease inhibitors.

## Introduction to Activity-Based Probes (ABPs)

Proteases represent a diverse class of enzymes that cleave proteins into peptides and finally to amino acids, thereby controlling the proteome to a steady-state level in the organism. Cysteine-, serine-, and threonine proteases utilize the corresponding amino acid residue to hydrolyze the scissile peptide bond, whereas aspartic, glutamic, and metalloproteases use their active site residues to activate water molecules to perform a nucleophilic attack of the carbonyl group at the carbon atom within the peptide bond. Activity-based probes (ABPs) are applied to detect the proteolytic activity of proteases and are referred to as activity-based protein profiling (ABPP). In general, ABPs contain three essential elements: a warhead, which is often an electrophilic group that reacts with the active site amino acid residue of the protease and results in an irreversible covalent binding; a recognition sequence, a peptide linker that matches the substrate specificity of the protease; and a reporter tag for detection of the protease-ABP complex (Oleksyszyn and Powers, 1989, Oleksyszyn and Power, 1991; Abuelyaman et al., 1994; Blum et al., 2009; Sienczyk and Oleksyszyn, 2009; Chakrabarty et al., 2019; Breidenbach et al., 2020; Kahler et al., 2020).

## ABPs: Diverse Warheads, Tags, and Applications

Several classes of warheads are incorporated into ABPs. The electrophilic groups are as follows: diphenylphosphonate, fluorophosphonate, and 4-chloroisocoumarin for targeting serine proteases; acyloxymethyl ketone, alkyne, α, β-unsaturated ketone, aza-epoxide, diazomethylketone, epoxysuccinate, halomethylketone, phenomethylketone, and vinylsulfone for targeting cysteine proteases; benzophenone and diazirine for targeting aspartic as well as metalloproteases; and epoxyketone and vinylsulfone for targeting threonine proteases. These are summarized in detail (Cravatt et al., 2008; Sanman and Bogyo, 2014; Willems et al., 2014). Highly selective ABPs allow detection of single active protease species and are synthesized by different strategies, such as 1) the incorporation of both non-natural and natural amino acids in the recognition sequence by using the so-called hybrid combinational substrate library (HyCoSuL) approach; 2) a combination of probes with antibodies building a hybrid system, activity-based ELISA, and activity-dependent proximity ligation; as well as 3) adding amino acid residues near the protease active site to bind the chemical probe with increased specificity (Kasperkiewicz et al., 2014; Sanman and Bogyo, 2014; Chakrabarty et al., 2019). There are several types of reporter tags, fluorescent dyes, isotopes, and biotin, which are detected by various techniques as described in the following section.

Fluorescent tags include boron-dipyrromethene- (BODIPY), cyanine-, fluorescein-, and rhodamine-type dyes which can be detected by fluorescence scanning of SDS-PAGE gels or *in vivo* fluorescence imaging (Hughes et al., 2016; Chen et al., 2017; Schulz-Fincke et al., 2018a). Furthermore, quenched fluorescent ABPs (qABPs) contain a warhead with a leaving group linked to a quenching moiety that is released from the probe upon reaction and subsequently generates a light emission from the qABP-protease complex (Serim et al., 2015; Edgington-Mitchell et al., 2017; Liu et al., 2019). Alternatively, qABPs can contain a warhead conjugated with a fluorophore where the emission of fluorescence is increased after the target protease is labeled. This mechanism is due to the photoinduced electron transfer effect (PeT) (Hong et al., 2017). The first qABPs for serine proteases consist of a mixed alkyl aryl phosphonate and are linked to a succinimide derivative of the QSy7 (fluorescent quencher) coupled to the tyramine leaving group. This component utilizes tetramethylrhodamine (TAMRA) as a fluorescent tag, isopropyl substituent as a spacer, *p*-guanidino-phenyl substituent to mimic an arginine side chain at the P1 position (P1 arginine mimic probe), and has been shown to strongly label trypsin-like proteases (trypsin and urokinase plasminogen activator), whereas a valine at P1 (P1 valine probe) labels neutrophil elastase (Serim et al., 2015).

Radioactive isotope-based tags, including ^125^I, ^127^I, ^18^F, ^111^In, and ^64^Cu, can be detected by the following techniques: magnetic resonance imaging (MRI), computed tomography (CT) imaging, positron emission tomography (PET), and single-photon emission computed tomography (SPECT) (Chakrabarty et al., 2019). Biotin tags, on the other hand, can be used to visualize proteolytic activity in a Western blot-based assay or can be used to facilitate a pull down of the respective proteases by immobilizing avidin resin and followed by liquid chromatography with tandem mass spectrometry (LC-MS/MS) analysis to identify the protease (Li et al., 2013; Mangold et al., 2018; Gruba et al., 2019).

ABPs utilizing 7-methoxycoumarin-4-yl-alanine as a reporter tag, peptide linker, either Leu-Lys-Ala-Ala (papain) or Pro-Leu-Phe-Ala-Glu-Arg (calpain), and thioamide leucine analogs as a quencher have demonstrated their applicability for the study of protease activity (Goldberg et al., 2014). Interestingly, the qABP BMV109, consisting of a Cy5 tag, spacer, phenoxymethyl ketone electrophile, and a sulfo-QSY21 quencher, selectively targets cathepsin B (CatB), L, S, and X, which are found to be overexpressed in certain cancers. Thus, BMV109 can be applied to identify tumor cells in tissues (Withana et al., 2016a). Additionally, advances were made in combining optical and PET/CT imaging applications. A modification of BMV109, which targets CatB, S, and L, led to a dual labeled ABP: BMV101. This probe contains a Cy5 for optical detection and a NOTA chelator for PET/CT applications and was used in a bleomycin mouse model investigating lung fibrosis (Withana et al., 2016b). Likewise, iodinated nanoscale ABPs (IN-ABPs) consisting of a polyamidoamine (PAMAM) dendrimer with an iodine tag and Cy5 fluorophore, carbobenzoxy–phenylalanine–lysine as the recognition element for CatB and CatL, and AOMK representing the warhead enables the detection of tumor cells using X-ray CT imaging (Gaikwad et al., 2018). Moreover, the ABP GB137, incorporating a Cy5 tag, Lys-Phe-Cbz at P1–P3 sites, with a reactive AOMK group, and QSY21 quenching moiety is capable of tracking CatB, L, and S activity within inflammatory macrophages and atherosclerotic plaques by using fluorescent molecular tomography (FMT) (Abd-Elrahman et al., 2016). Other ABPs are generated for targeting the serine proteases matriptase and matriptase-2; these components contain the diphenyl phosphonate warhead, two guanidinophenyl moieties as arginine mimetics, and coumarin (aromatic lactone) as the fluorophore that can be detected by direct in-gel or HPLC fluorescence scanning (Haussler et al., 2016; Haussler et al., 2017; Breidenbach et al., 2020).

Another example of an ABP is the acyloxymethyl ketone (AOMK) component, a cysteine reactive electrophile, including Gln/Gln(Me)_2_-Leu-Tle-Abu at the P1–P4 position for covalent labeling of the SARS-CoV-2 main protease (M^pro^) and harboring a TAMRA fluorophore or biotin as a detection tag (van de Plassche et al., 2020). ABPs containing an acetylated peptide sequence with a vinyl sulfone as an irreversible reactive group and a polyethylene glycol as a linker with a N-terminal biotin tag or the cyanine 5 dye (Biotin-PEG(4)-Abu-Tle-Leu-Gln-VS and Cy5-PEG(4)-Abu-Tle-Leu-Gln-VS) are other examples of ABPs able to detect the M^pro^ of SARS-CoV-2 (Rut et al., 2020). These ABPs allow further development of anti-SARS-CoV-2 drugs or can be used as diagnostic tools.

Overall, the addition of ABPs to noninvasive optical and PET/CT imaging leads to an improvement of drug discovery, tumor categorization (Gaikwad et al., 2018), and investigation of disease progressions as well as resolution of the respective mechanism (Withana et al., 2016b).

## ABPs and Their Applications to Analyze Serine Proteases

Neutrophils are part of the innate immune system and are responsible for the first line of defense by migrating from the blood vessel to the site of infection to combat invading pathogens. When activated, neutrophils release several mediators, including neutrophil serine proteases (NSPs) such as cathepsin G (CatG), protease 3 (PR3), neutrophil elastase (NE), and neutrophil serine protease 4 (NSP4) (Korkmaz et al., 2008). The ABP, composed of the recognition sequence Bt-Val-Tyr-Asp-nVal^P^(O-C_6_H_4_-4-Cl)_2_, which is an α-aminoalkylphosphonate diaryl ester derivative and harbors a biotin tag, labels PR3 (Grzywa et al., 2019). Alternatively, the sulfonyloxyphthalimide moiety warhead with a peptide linker and a coumarin fluorophore for in-gel fluorescent detection can be used to detect porcine pancreatic elastase. The mechanism of elastase detection involves its nucleophilic attack of the active site serine amino acid residue to the carbonyl carbon of sulfonyloxyphthalimide and results in the application of Förster resonance energy transfer (FRET) (Schulz-Fincke et al., 2018b). Two specific small-molecule ratiometric NE monitoring reporters based on energy transfer were synthesized to detect soluble NE and neutrophil secreted NE to monitor cell surface-associated NE activity (Gehrig et al., 2012). Furthermore, the hybrid combinatorial substrate library positional scanning approach was applied to generate highly selective substrates and ABPs for profiling of individual active NSPs. This method includes the synthesis of a combinatorial library which incorporates a hybrid of natural and unnatural amino acids (increase the specificity) into the substrate followed by screening with the respective protease (Kasperkiewicz et al., 2014; Kasperkiewicz et al., 2015; Kasperkiewicz et al., 2017). In order to overcome extensive washing steps of cells or long clearance time removing unbound ABP in biological samples, an alkyl aryl phosphonate-based qABP was synthesized which binds trypsin and the urokinase plasminogen activator by incorporating the P1 arginine mimic element into the qABP or valine in P1 for neutrophil elastase labeling. After the covalent binding of ABPs to the serine protease, the fluorescent quencher is released and fluorescence of TAMRA can be detected (Serim et al., 2015). qABPs can be further improved by incorporating a multibranched fluorescence quencher to eliminate cell toxicity and background fluorescent levels (Craven et al., 2018).

In the following sections, we will mainly summarize CatG and its respective ABPs visualized by different techniques.

### The Source of CatG: Neutrophils

Neutrophils are a type of immune cell containing cytosolic granules of various types, compromising proteases and other mediators assigned to initiate an immune response (Korkmaz et al., 2008). The cytosolic granules belong to lysosomal-related organelles and are classified as primary (azurophil), secondary, and tertiary granules. Serine proteases, including CatG, are abundant in primary granules and can be released to the extracellular space to initiate an innate as well as an adaptive immune response. Proteases of the cysteine-, aspartic-, and matrix metalloprotease (MMP) class are also essential and reside in diverse granules (Benarafa and Simon, 2017). Lactoferrin (LF) is abundant in the secondary granules. In general, LF is not only capable of binding free ferric iron (Kruzel et al., 2007) but also exhibits serine protease activity (Hendrixson et al., 2003). Besides preventing bacterial invasion, LF can interfere with glioblastoma cell growth (Arcella et al., 2015), protects against oxidative stress (Kruzel et al., 2013), is critical for maintaining physiologic homeostasis, controls the inflammatory response (Kruzel et al., 2017), and is able to increase the catalytic activity of CatG (Eipper et al., 2016). Tertiary granules, in turn, contain mainly MMP-9 and ficolin, which are instantly released by neutrophils into the environment after stimulation (Rorvig et al., 2009; Khokha et al., 2013).

### Biochemical Properties of CatG

The catalytic triad of CatG possesses histidine (H57), aspartate (D102), and serine (S195) amino acid residues and is located between the two homologous asymmetric β-barrels of the three-dimensional structure of human CatG. Thereby the oxygen atom of the hydroxyl group of S195 performs a nucleophilic attack on the carbonyl carbon atom of the scissile peptide bond (Hof et al., 1996; Korkmaz et al., 2010). Human CatG has a preference to hydrolyze the peptide bond after K and R (trypsin activity); F, Y, and W (chymotrypsin activity); L (leuase activity); M (metase activity); and N at the P1 position. Furthermore, CatG prefers P at P2; E at P3; D and T at P4; and I, A, and S at the alternate subsite P1′ as well as D and E at the P2′ subsite (Wysocka et al., 2007; Raymond et al., 2010; Swedberg et al., 2017; Nguyen et al., 2018; Thorpe et al., 2018).

### Application of ABP to Analyze Proteolytic Activity in Biological Samples

In general, CatG is involved in defending the organism from invading pathogens. However, in distinct circumstances, it was found that CatG is not regulated properly, for instance, CatG is increased in photoaged human skin (Zheng et al., 2011), chronic inflammatory pain (Liu et al., 2015), and rheumatoid arthritis tissue (Adkison et al., 2002; Hu and Pham, 2005; Miyata et al., 2007) as well as in peripheral blood mononuclear cells (PBMCs) from type 1 diabetes mellitus (T1D) (Zou et al., 2011).

Bt-P1,5D-Suc-Val-Pro-Phe^P^(OPh)_2_, which is also denoted as MARS116, is an ABP that can detect CatG activity and was synthesized based on the work of Oleksyszyn and colleagues, where preferable amino acids of CatG within the P1 and P2 positions were incorporated into the peptidyl diphenyl phosphonate (Oleksyszyn and Powers, 1991). In addition to increasing the sensitivity of the ABP, a spacer was included resulting in detection of a low amount of CatG and a biotin tag for visualization in a Western blot-based assay (Zou et al., 2012).

**FIGURE 1:**
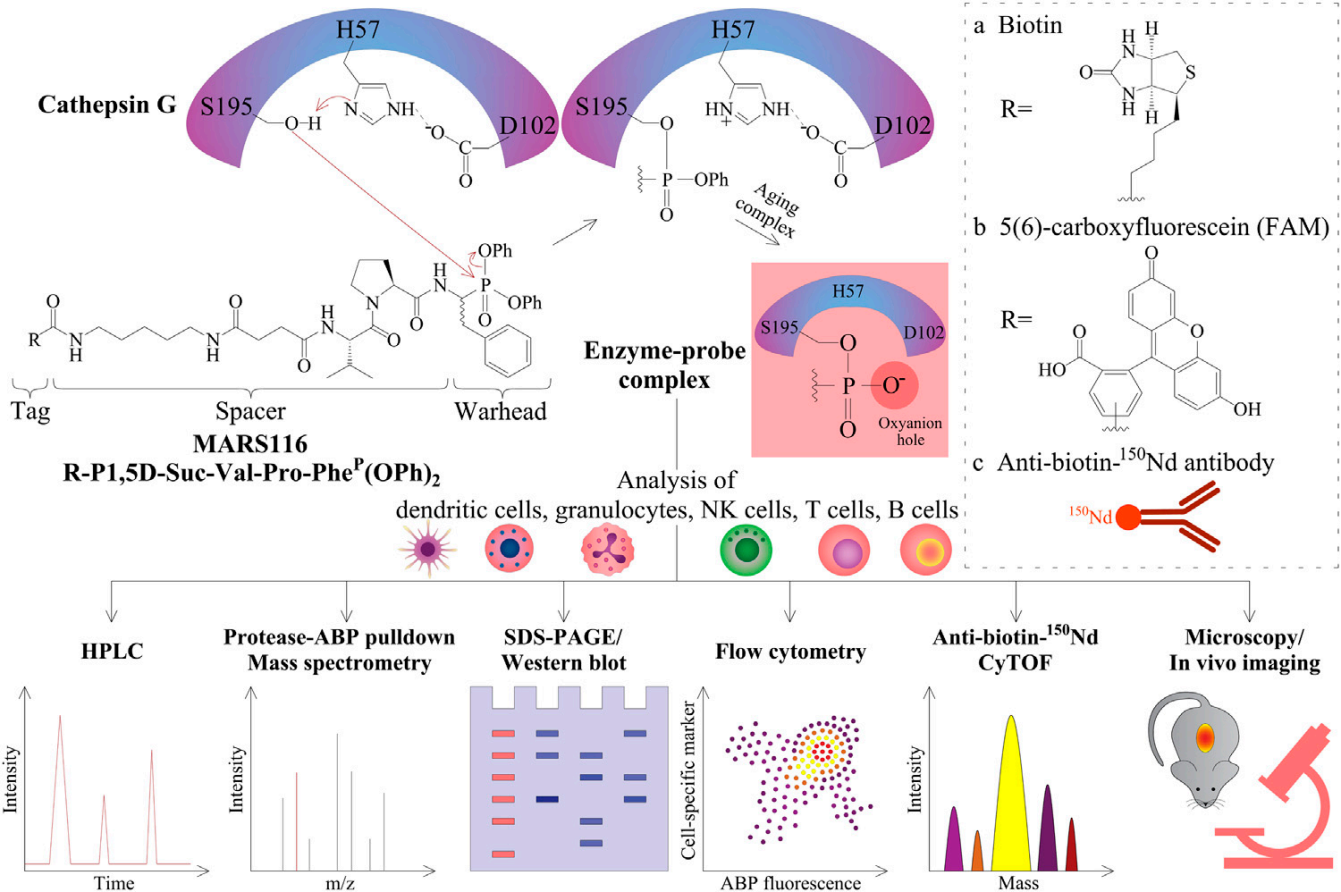
Peptidyl diphenyl phosphonates and their applications. The three amino acid residues of H57, D102, and S195 form the catalytic triad of CatG. After substrate binding to this active site, the nitrogen atom of H57 abstracts a proton in a general acid–base catalysis. Thereby, the oxygen atom from the serine amino acid side chain S195 (alkoxide ion) becomes a strong nucleophile and attacks the partially positively charged phosphorous atom of the diphenyl phosphonate warhead of MARS116. The two electronegative phenoxy groups further enhance the electrophilicity of the phosphorous atom. As a result of the nucleophilic attack of S195, the phenoxy group is released and the oxygen atom of the S195 side chain binds covalently to MARS116. The second phenoxy group leaves the active site in a time-dependent process where the remaining negatively charged oxygen atom stays in the oxyanion hole. R can be either a biotin **(A)**, a fluorophore **(B)**, or an ^150^Nd conjugated antibody reactive toward biotin **(C)** for detection of CatG by different approaches, such as HPLC with a fluorescence detector, pull-down and LC-MS/MS analysis, SDS-PAGE and Western blot, flow cytometry, CyTOF, fluorescence microscopy, and possibly imaging.

Regarding the mechanism of action to profile CatG, the phosphorus atom of the phosphonate warhead is attacked by the nucleophilic oxygen atom of the active site serine side chain, S195, which provokes the exit of the phenoxy leaving group (Figure 1). As a result, the phosphonate binds covalently to the S195 side chain, and the “aging complex” of CatG-MARS116 loses the second phenoxy leaving group, where the negatively charged oxygen atom stays and occupies the oxyanion hole of the catalytic center. This mechanism can be used to detect CatG in cell lysates by a Western blot-based assay since MARS116 contains biotin (Zou et al., 2012; Grzywa and Sienczyk, 2013; Kahler et al., 2020). Moreover, a 96-well ELISA-based colorimetric active site-specific immunoassay (CASSIA) (Mann et al., 1990) was utilized to assess CatG activity by a MARS116 high-throughput assay. In this assay, cell lysate from CatG or CatG containing PBMCs was labeled by MARS116, and the CatG-MARS116 complex was transferred to a CatG recognizing specific antibody precoated 96-well ELISA plate for CatG activity detection *via* streptavidin-HRP and the respective substrate, 3,3′,5,5′-tetramethylbenzidine (Zou et al., 2012).

MARS116 is also appropriate to be applied to detect CatG activity on the surface of cells and has been demonstrated by fluorescence microscopy (Grzywa et al., 2014). The advantage of using flow cytometry is that cell separation from a mixture of cells can be circumvented and the proteolytic activity can be determined extra- and intracellularly by ABPs directly. The application of ABPs in flow cytometry was first performed to detect cysteine-aspartic proteases (caspases) by using a caspase inhibitor attached to fluorescein isothiocyanate (FITC) (Pozarowski et al., 2003) and followed by a more selective non–peptide-based ABP to detect the cysteine protease CatS (Verdoes et al., 2012). In order to use ABPs to analyze serine proteases in flow cytometry, we employed MARS116 in PBMC samples to detect active CatG in diverse immune cell subsets by avidin-FITC (Penczek et al., 2016) as well as by anti-biotin-^150^Nd metal isotope which was analyzed by the so-called mass cytometry by time-of-flight (CyTOF) (Gärtner et al., 2020). Additionally, a direct 5(6)-carboxyfluorescein (FAM) conjugated MARS116 version was synthesized (MARS116-FAM) for intracellular detection of CatG by flow cytometry (Schroeder et al., 2020).

Remarkably, metal-tagged, time-of-flight activity-based probes (also called TOF probes) were generated by incorporating an N-terminal tetracarboxylic acid (DOTA)-chelated stable isotope of lanthanoids with a C-terminal acyloxymethylketone (AOMK) attached to the diphenyl phosphonate to simultaneously detect four different protases, CatB, CatL, asparagine-endoprotease (AEP also called legumain), and neutrophil elastase, in cell lines and PBMCs by using CyTOF as well as imaging mass cytometry (IMC) (Poreba et al., 2020). TOF probes, ^159^Tb CatB, ^175^Lu CatL, ^158^Gd legumain, and ^159^Tb NE, were applied to detect CatB, CatL, legumain, and NE in immune cells using CyTOF, and high legumain was found in B cells of one donor indicating infection or cancer (Poreba et al., 2020). Indeed, the proteolytic content of legumain (AEP) was compared between primary human B cells isolated from PBMCs (primary CD22^+^ B cells) and the EBV-transformed B cell line and found high levels of AEP in the EBV-transformed B cell line in contrast to primary CD22^+^ B cells. In primary CD22^+^ B cells, AEP expression and activity was absent analyzed by RT-PCR, turnover of AEP substrate, and digest experiments with lysosomal fractions using a model antigen myelin basic protein (Burster et al., 2004).

CyTOF is a novel cytometry method that enables the simultaneous detection of up to 100 surface and intracellular antigens in one approach (Cheung and Utz, 2011; Kay et al., 2016). Although recent developments in the field of polychromatic flow cytometry have been made, which support analysis up to 30 parameters, it has been shown that background error and the inevitable issue of compensation due to the spectral overlap of acquired samples contributes to variability during automated analysis of the data set using computational approaches such as t-distributed stochastic neighboring embedding (tSNE) (Mazza et al., 2018). Differential background distribution due to background calculation as well as spectral overlap is not an issue in CyTOF, since the CyTOF approach shifts from the use of fluorochrome conjugated antibodies to transition metal isotopes conjugated to antibodies. These metal isotopes consist of rare earth elements as well as noble and post-transition metal isotopes (Han et al., 2018). Subsequent to the staining, CyTOF vaporizes the cells and measures the metal isotopes with a time-of-flight mass spectrometer instead of exciting the fluorochromes with a laser (Bendall et al., 2011). It is unlikely that CyTOF will replace regular flow cytometry due to the destruction of the cells during measurement (it is not possible to sort cells) and the reduced flow rate (Gadalla et al., 2019). The opportunity to add ABPs to CyTOF (Gartner et al., 2020; Poreba et al., 2020) offers an advanced tool for deep profiling of cells for their extracellular as well as intracellular proteolytic activity of proteases and increases the analytical and mechanistical resolution of the method even further.

### Extracellular Study of CatG Activity by ABPs

**FIGURE 2:**
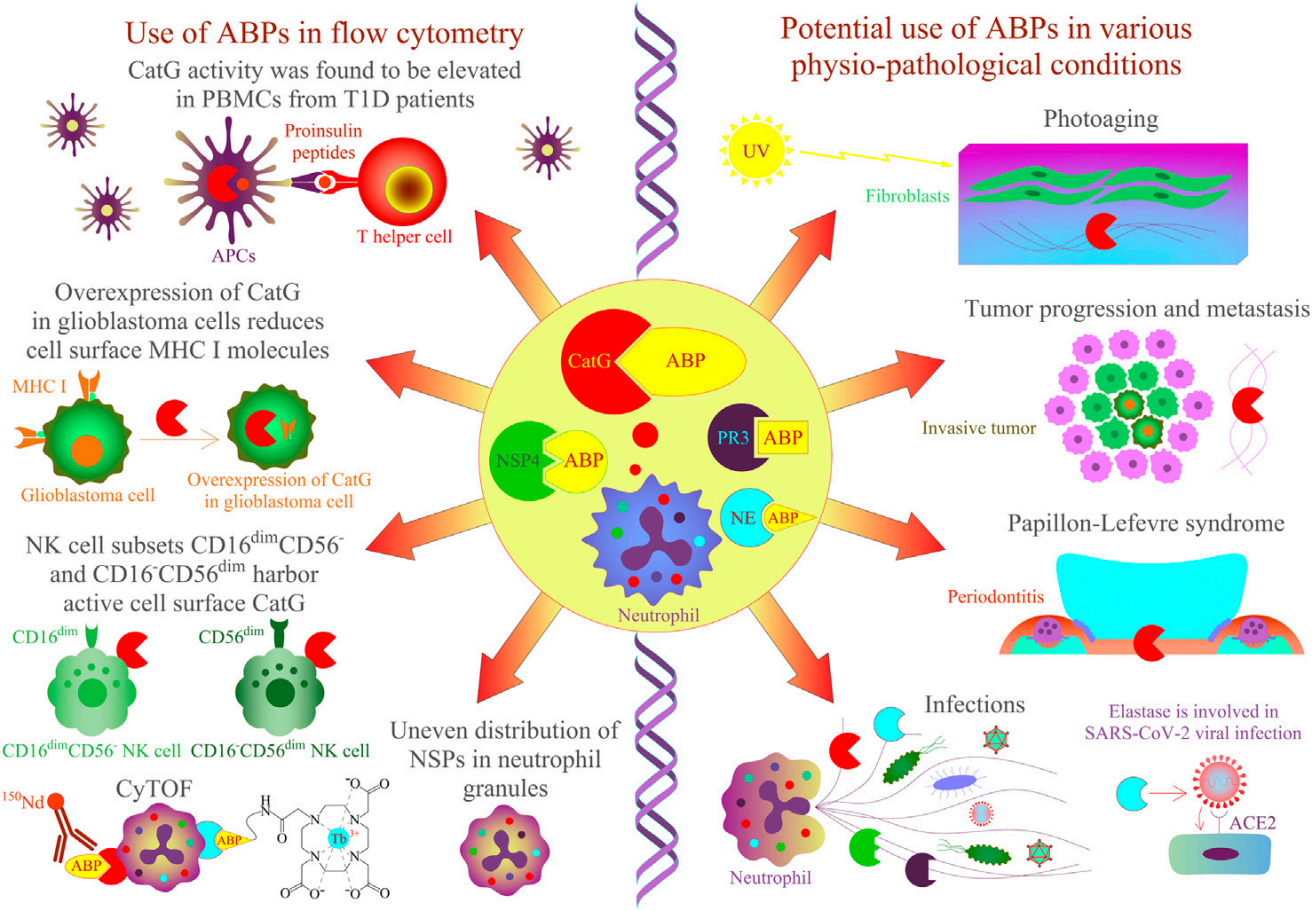
Application of ABPs for NSPs in cells and tissues by flow cytometry approaches. CatG-specific ABPs have been shown to detect protease activity in different biological samples. On the left panel, CatG is involved in proinsulin processing, and its activity was found to be elevated in PBMCs from T1D patients by using an activity-based probe (DAP22c) and T cell activation was assessed by flow cytometry (Zou et al., 2011). NK cell subsets CD16^–^CD56^dim^ and CD16^dim^CD56^–^ harbor active cell surface CatG (Penczek et al., 2016) and CD16^+^CD56^–^, eosinophils, and neutrophils by using MARS116 in CyTOF analysis (Gartner et al., 2020). The overexpression of CatG in glioblastoma cells was demonstrated to downregulate cell surface MHC I molecules, and CatG activity was verified by using the MARS116 probe (Palesch et al., 2016). NSPs, including CatG, are unevenly located in the granules of neutrophils (Kasperkiewicz et al., 2017), and neutrophil elastase can be detected in various immune cells by CyTOF (Poreba et al., 2020). On the right panel, CatG-specific ABPs could be used to investigate protease activity in various physiopathological conditions such as photoaged human skin, cancer, Papillon–Lefevre syndrome, inflammation, and infection [Hu et al., 2020; Korkmaz et al., 2010; Burster et al., 2020)].

Besides addressing the question about functional properties of cell surface CatG, it remains vague as to why ABPs should be applied to detect CatG on the cell surface of living cells. Regulation and inhibition of CatG of distinct cell subsets, resident in the blood stream or different tissues, can be evaluated by ABPs (Figure 2). For instance, the efficiency of drugs (serine protease inhibitors) to inhibit the proteolytic activity of CatG of infiltrating immune cells into the inflamed tissue can be monitored by using multicolor cytometry (Penczek et al., 2016) or more advanced by CyTOF, which opens the possibility for multiplexed profiling of different cells. In this regard, we have shown the ability to detect the proteolytic activity of CatG on several immune cells, including neutrophils, eosinophils, and NK cell subsets in an immune phenotyping approach. Thereby, the gating strategy is based on DNA staining with ^191^Ir and ^193^Ir, which are cationic nucleic acid intercalators to discriminate living cells from dead cells and single nucleated cells from doublets. CatG activity was determined by the cell surface expression of CD3^−^CD20^−^CD56^−^CD66b^+^CD16^+^ on neutrophils, CD3^−^CD20^−^CD56^−^CD66b^+^CD16^−^ on eosinophils, and CD3^−^CD14^−^CD20^−^CD56^var^CD16^var^ on NK cells. Moreover, the use of ABPs can be applied to any given phenotyping panel and enables the detection of CatG activity on the cell surface of blood as well as on tissue-derived cells (Gärtner et al., 2020).

Generally, levels of CatG on the cell surface of neutrophils (Owen et al., 1995), B cells, NK cells (Delgado et al., 2001), platelets (Selak, 1994), CD39^+^ T regulatory cells (Tregs), double positive CD4^+^CD8^+^ T cells, T helper cells 9, and T helper cells 22 (Penczek and Burster, 2019) are detected, but CatG is absent on resting CD8^+^ T cells (Yamazaki and Aoki, 1997; Delgado et al., 2001). Interestingly, CD4^+^ T cells, NK cells, B cells, and CD8^+^ T cells can bind CatG exogenously (Yamazaki and Aoki, 1997). The advantage of MARS116 in flow cytometry is that CatG activity at the cell surface of distinct immune cell subsets with different functional roles in an immune response can be analyzed. For instance, we found that the NK cell subsets CD16^−^CD56^dim^ and CD16^dim^CD56^-^ harbor active cell surface CatG (Penczek et al., 2016). NK cell development undergoes differentiation from naive CD56^bright^ to mature CD56^dim^ expressing NK cells. While CD56^bright^ NK cells secrete immunoregulatory cytokines/chemokines after activation, CD56^dim^ NK cells are effective cytotoxic NK cells (Bjorkstrom et al., 2010; Juelke et al., 2010; Stabile et al., 2017). There has been speculation that activated CD16^−^CD56^dim^ and CD16^dim^CD56^-^ kill the target cell by secreting perforin and granzyme B. CatG on the cell surface could protect from self-lysis by inactivating both perforin and granzyme B in a proteolytic manner (Burster et al., 2020).

Application of ABPs in flow cytometry or the next generation of flow cytometry CyTOF for multiplexed profiling of up to 100 markers can be used for sufficient detection and inhibition of protease activity. The addition of such an ABP to a high dimensionality phenotyping panel would add valuable insights into its function and strengthen the application of CyTOF in the laboratory and in clinical use. This would meet the effort to gain mechanistic insights even with a limited clinical material and would fit well to a standardized CyTOF workflow for system-level biomarker discovery. Moreover, the principle of assessing the proteolytic activity of CatG by using a biotin-coupled ABP to detect proteolytic activity at the cell surface (Gartner et al., 2020) or more advanced by using ABPs with N-terminal tetracarboxylic acid-chelated stable isotope of lanthanoids (Poreba et al., 2020) could be applied to the progressive field of CyTOF and optical imaging by combining multidimensional single-cell analysis with histological stratification. An example of this combination is the Hyperion imaging mass cytometry technology that offers spatially resolved single-cell analysis using mass tagged antibodies (Jackson et al., 2020). Including ABPs for proteases would increase the analyzing capacity of tumor cells infiltrating the healthy tissue by detecting their proteases and the possible inhibition with protease inhibitors. The proof of principle of ABPs of optical imaging has been performed previously in a comparative study, which showed that the use of ABPs offers an excellent alternative to large polymer-based molecules, leading to a brighter signal intensity (Blum et al., 2009) or *in vivo* imaging of thrombin *via* ABP (Jaffer et al., 2002).

In conclusion, novel selective protease inhibitors can be tested by flow cytometry, CyTOF, and optical imaging to investigate whether such inhibitors are effective against the proteolytic activity of CatG and other proteases at the cell surface. Moreover, analysis of CatG activity can contribute to the observation of pathogenesis of diseases from complex biological samples with a vast panel of cell markers in a single-cell resolution. In the next paragraph, we will focus on the detection and functional properties of intracellular CatG.

### Intracellular Study of CatG Activity by ABPs

The advantage of flow cytometry is that a mixture of cells within samples or cell subsets is gated and analyzed for active proteases. Additionally, cells treated with drugs, cytokines, or other mediators can be simultaneously monitored for their activation status by using different activation markers (Burster et al., 2020; Kahler et al., 2020).

Intracellular proteolytic active NSPs can be detected by ABPs in a flow cytometry approach and reflect an additional method to fluorescence microscopy for colocalization of active proteases. Precisely, imaging flow cytometry, which analyzes intensity, location, and colocation of, for instance, ABPs are used to detect and analyze the localization of NSP activity within cell compartments by applying a bright field illuminator, a laser, and the respective software (imaging data exploration and analysis software, IDEAS). Thereby, the authors found a nonoverlapping punctate distribution of active CatG, PR3, NE, and NSP4, which means that these NSPs are unevenly located in the granules of neutrophils (Kasperkiewicz et al., 2017).

It was demonstrated that MARS116 conjugated with biotin is not suitable for detection of intracellular CatG of biological samples, since the necessary second labeling step with avidin-FITC binds unspecifically or to intracellular biotinylated proteins. Thus, direct conjugation of FAM to MARS116 results in the MARS116-FAM component, which overcame the obstacle of unspecific binding of avidin-FAM and can be applied to detect intracellular CatG activity by flow cytometry. Certainly, MARS116-FAM allows a faster labeling procedure, and, subsequently, extra staining as well as washing steps can be circumvented. Besides the procedure of MARS116-FAM to detect intracellular CatG activity, this ABP is also suitable to detect CatG activity immediately after activity-based protein profiling (ABPP) in SDS-PAGE gels (Schroeder et al., 2020).

Generally, Tregs are important for maintaining an immune response, immune homeostasis, and tolerance and are subdivided into thymus-derived natural Tregs, induced Tregs, and peripheral Tregs (Kanamori et al., 2016). The cell surface molecule CD39, expressed on Tregs, hydrolyzes extracellular ATP and ADP to generate AMP, which is further transformed to adenosine by the additional cell surface molecule CD73. Adenosine itself docks to the cell surface A2A receptor of T cells and effectively inhibits effector T cell function (Borsellino et al., 2007; Deaglio et al., 2007). A defined subset of Tregs present in the human peripheral blood is CD39^+^ which expresses CatG on the cell surface; however, this protease is absent on CD39^−^ Tregs suggesting different functional properties (Penczek and Burster, 2019). The difference of CatG distribution between the two subsets can be partly explained by the finding that higher levels of intracellular CatG activity were found in CD39^+^ Tregs in contrast to CD39^−^ Tregs explaining the absence of CatG on the cell surface of CD39^−^ Tregs. These results were determined by analyzing PBMCs and utilizing MARS116-FAM in flow cytometry (Burster et al., 2020; Schroeder et al., 2020).

ABPs are also crucial for the detection of proteolytic activity in samples from patients. For instance, it was demonstrated that CatG activity, analyzed by MARS116 in a Western blot-based assay, was increased in PBMCs from T1D patients in comparison to PBMCs isolated from healthy donors, and CatG was responsible for generating insulin-derived T cell epitopes supporting the autoimmune event by activating autoreactive T cells (diabetogenic T cells) (Zou et al., 2011). It would be of interest to analyze the precise immune cells or immune cell subsets where high CatG activity might be present in order to generate specific modulators interfering with insulin-dependent antigen processing and presentation to diabetic T cells. This could be performed by the application of ABPs in a flow cytometry approach.

ABPs can not only contribute to the analysis of CatG activity in tissue or cells from pathological compared to healthy samples, but are is also suitable to be used to monitor pathogenesis where CatG is indicated as a marker or to identify proteolytic inhibition by selective inhibitors for CatG.

## Conclusion

ABPs can be used for studying protease enzymology, drug development, and for diagnostics in diverse cells. For instance, selective ABPs to determine CatG activity in cells can be performed by different and novel methodologies, including gel electrophoresis, optical imaging, fluorescence microscopy, conventional flow cytometry and imaging flow cytometry, and CyTOF. The latter three are potent methods to distinguish immune cell subsets in a cytometry-based approach. Generation of selective ABPs for proteases to be analyzed in flow cytometry can be applied for biological samples to determine the proteolytic activity or whether proteases are controlled properly by administration of effective inhibitors.

## Abbreviations

ABP, activity-based probe; ABPP, activity-based protein profiling; AOMK, acyloxymethyl ketone; APCs, antigen-presenting cells; Bt, biotin; BODIPY, fluorescent tags include boron-dipyrromethene; Cat, cathepsin; CatG, cathepsin G; CyTOF, mass cytometry by time-of-flight; FRET, Förster resonance energy transfer, FITC, fluorescein isothiocyanate; LF, lactoferrin; MARS116-Bt, Marcin Sienczyk 116-biotin activity-based probe (Bt-P1,5D-Suc-Val-Pro-Phe^P^(OPh)_2_); MARS116-FAM, 5(6) carboxyfluorescein conjugated to MARS116; M^pro^, main protease; MHC, major histocompatibility complex; MMPs, matrix metalloproteinases; NE, neutrophil elastase; NK cells, natural killer cells; NSP4, neutrophil serine protease 4; PBMCs, peripheral blood mononuclear cells; PR3, proteinase 3; P1,5D, pentane-1,5-diamine; qABP, quenched fluorescent ABPs; RAS, renin angiotensin system; tSNE, t-distributed stochastic neighboring embedding; TGF-β, transforming growth factor beta; Th, T helper cells; TNF-α, tumor necrosis factor α; Tregs, T regulatory cells
